# Effects of ClpP protease on biofilm formation of *Enterococcus faecalis*

**DOI:** 10.1590/1678-7757-2020-0733

**Published:** 2021-03-01

**Authors:** Ying FENG, Hongyuan WANG, HE Lu, LIU Yi, LI Hong

**Affiliations:** 1 Department of Endodontics School of Stomatology Capital Medical University China Department of Endodontics, School of Stomatology, Capital Medical University.; 2 Affiliated Stomatology Hospital Guangzhou Medical University China Affiliated Stomatology Hospital of Guangzhou Medical University.; 3 Capital Medical University Beijing Key Laboratory of Tooth Regeneration and Function Reconstruction School of Stomatology Capital Medical University China Laboratory of Tissue Regeneration and Immunology and Department of Periodontics, Beijing Key Laboratory of Tooth Regeneration and Function Reconstruction, School of Stomatology, Capital Medical University.

**Keywords:** Enterococcus faecalis, ClpP protease, Biofilm, Growth

## Abstract

*Enterococcus faecalis* (*E. faecalis*), one of the main pathogens responsible for refractory periapical periodontitis and nosocomial infections, exhibits markedly higher pathogenicity in biofilms.

**Objectives:**

Studies have shown that caseinolytic protease P (ClpP) is involved in biofilm formation. However, to date, few studies have investigated the role of ClpP in the survival of *E. faecalis*, and in enhancing biofilm formation. Therefore, we investigated the role of ClpP in the formation of *E. faecalis* biofilms.

**Methodology:**

In our study, we used homologous recombination to construct clpP deleted and clpP complement strains of *E. faecalis* ATCC 29212. A viable colony counting method was used to analyze the growth patterns of *E. faecalis*. Crystal violet staining (CV) and confocal scanning laser microscopy (CLSM) were used to characterize biofilm mass formation and scanning electron microscopy (SEM) was used to observe the biofilm microstructure. Data was statistically analyzed via Student’s t-test or one-way analysis of variance (ANOVA).

**Results:**

The results exhibited altered growth patterns for the clpP deletion strains and depleted polysaccharide matrix, resulting in reduced biofilm formation capacity compared to the standard strains. Moreover, ClpP was observed to increase bioﬁlm formation in *E. faecalis*.

**Conclusion:**

Our study shows that ClpP can increase bioﬁlm formation in *E. faecalis* and emphasizes the importance of ClpP as a potential target against *E. faecalis*.

## Introduction

*Enterococcus faecalis* (*E. faecalis*) is one of the primary etiologic pathogens of refractory periapical periodontitis, secondary root canal infections, and pulp infection. It is also the main pathogen of nosocomial infections, such as bacterial endocarditis and meningitis.^1-2^ The ability of *E. faecalis* to form biofilms directly determines its survival, adaptability to the environment and pathogenicity. It was reported that more than 40% of *E. faecalis* clinical isolates can form biofilms.^3^*E. faecalis* is 100–1000 times more pathogenic in biofilms than in the plankton state,^4^ since biofilms provide protection against environmental stress, including lacks of nutrients, high alkalinity and antibiotics.^5^ In the field of Endodontics, the medicaments used to remove bacteria from the root canal system primarily include calcium hydroxide and antibiotics. However, these agents are associated with poor efficiency and have been shown to cause adverse effects.^6^ Hence, there is currently a lack of effective medication capable of treating *E. faecalis*. Therefore, investigating the mechanism associated with *E. faecalis* biofilm formation is essential to inform the development of effective treatment methods.

Accordingly, the stress response of *E. faecalis* has become the subject of intensive research. The adaptability of *E. faecalis* to stress and cross-immunoprotectivity is associated with increased protein synthesis. However, except for a few stress-related proteins, such as Gls24, and the molecular chaperones, GroEL and DnaK, most proteins involved in the stress response remain unknown.^7-8^ Caseinolytic protease P (ClpP) is an ATP-dependent proteolytic enzyme that plays important roles in bacteria, parasites, and human mitochondria. Specifically, ClpP participates in the hydrolysis of misfolded and defective proteins, whereas *ClpP* is an important gene involved in biofilm formation.^9-18^

Recent studies have reported that ClpP exerts different effects on bacterial biofilm formation. However, the general consensus is that ClpP enhances biofilm formation, since the biofilm formation of *Streptococcus mutans, Actinobacillus pleuropneumoniae,* and *Bacillus subtilis* decrease when ClpP was mutated.^12-14^ Our previous study found that ClpP of *P. gingivalis* improved biofilm formation by regulating adhesion-related factors and the density-sensing system.^15^ Meanwhile, other studies have shown that ClpP inhibits biofilm formation. For example, studies reported that ClpP inhibits biofilm formation by regulating Sle1 and agr in *Staphylococcus aureus*.^16^ In fact, researchers hold different views on the role of *clpP* in the formation of biofilm for same bacteria. Shanks, et al.^17^ (2006) found that when *clpP* was mutated, the biofilm of *Pseudomonas aeruginosa* increased; however, Hall, et al.^18^ (2017) stated that *clpP* enhanced its ability to form biofilms.

To date, few studies have investigated the role of ClpP in the survival of *E. faecalis* or its ability to influence biofilm formation. *E. faecalis* has a unique ability to tolerate hypoxia, starvation, and high alkalinity in reinfected root canals making it challenging to treat with current antibiotics. Hence, identifying effective antibiotic targets is an urgent concern for this pathogen. In our study, we investigated the role of ClpP in the formation of *E. faecalis* biofilms. These results advance the current understanding on the role of ClpP in *E. faecalis* biofilm formation and may lead to novel antibacterial therapies in the future.

## Methodology

### Bacterial culture conditions and plasmids

The *E. faecalis* standard strains ATCC 29212 (Manassas, VA, USA) was cultured in brain heart infusion medium (BHI, Oxoid Deutschland GmbH, Wesel, Germany) in an anaerobic environment at 37°C. BHI plates contained v/v vitamin K, hemin, and 5 g yeast extract/L (Sigma Aldrich). *Escherichia coli* strains JM109 was grown in Luria-Bertani medium. Antibiotics were purchased from Sigma Chemical Co.

### Construction of deletion and complemented strains

**Figure 1:**
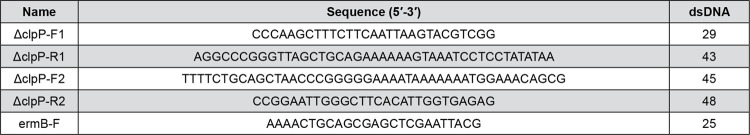
Primers used for PCR

Based on the strains ATCC 29212 chromosomal DNA sequence, primers for the *clpP* gene were designed (Figure 1). PCR was used to amplify the upstream and downstream homologous fragments, and the product was cloned into plasmid pUC18. Based on the *ermB* gene sequence of plasmid pJRS233, the *ermB* gene fragment was amplified and inserted into the recombinant plasmid pUC18-*ΔclpP*. pUC18-*ΔclpP*, pUC18-*ΔclpP-ermB*, and pUC18-*ΔclpP-ermB-clpP* were transformed into *E. coli* strains JM109 and selected for with ampicillin. The *clpP* deletion and complement plasmids were then transformed into *E. faecalis*. PCR and enzyme-cutting electrophoresis were used to identify the accuracy of the sequence, and high-expression strains were selected using media.

### Growth analysis of *E. faecalis*

Frozen *E. faecalis* ATCC29212 was added to 5 mL of BHI broth and cultured under anaerobic conditions at 37°C for 16 h. Next, 500 µL of bacterial solution (1.5 × 10^9^ CFU/mL) was added to 50 mL of BHI medium, from which 1 mL of bacterial solution was serially diluted and cultured under anaerobic conditions at 37°C. From 0 h the culture was observed every 2.5 h for a total of 25 h. The cultures were also observed every 12 h for a total of 61 h. Bacterial growth was recorded using the viable colony count method.

### Construction of the biofilm model

*E. faecalis* standard, mutant, and complementary strains were inoculated in BHI broth. After anaerobic culture at 37°C for 24 h, independent colonies were selected in logarithmic phase and cultured in BHI liquid medium at 37°C. The bacterial density was then adjusted to 10^5^CFU/mL. In a sterile environment, suitable size coverslips were placed in 12-well plates and 1 mL of *E. faecalis* suspension was added. One tablet was used for each strain. Plates were incubated at 37°C for 6, 12, 24, and 48 h. The experiments were repeated independently in triplicate for each time point and with each strain.

### Crystal violet staining

Crystal violet (CV) staining was performed to quantify the biofilm mass. The biofilms were constructed using 12-well polystyrene tablets. One tablet was used for each strain. The culture samples were fixed with 200 µL 1% paraformaldehyde for 15 min and washed three times with 200 µL phosphate buffered saline (PBS). Subsequently, 200 µL of 0.01% CV was added for 15 min, and 200 µL PBS was used for clearing. After mixing with 200 µL of ethanol and acetone for 5 min, the product was transferred to a 96-well plate and OD 570 readings were obtained for quantification. Three wells were used for each sample at each observation time point. The observation times were at 6, 12, 24, and 48 h. Each assay was repeated three times.

### Confocal scanning laser microscopy

Confocal scanning laser microscopy (CLSM) was performed to determine *E. faecalis* cell viability and to observe the attached bacterial areas. Each strain was cultured under anaerobic conditions at 37°C for 6, 12, 24, and 48 h. A total of 200 μL SYT09 and PI fluorescent dyes were added to samples in the dark for 15 min at 37°C. In addition, FITC-ConA and PI were used to stain the polysaccharide matrix. The processed biofilms were observed using the CLSM system (TCS SP5; Leica Microsystems, Germany). Live and dead bacteria emitted green and red fluorescein, respectively. An image-processing program (Leica Microsystems) was used for digital reconstruction.

### Scanning electron microscopy

Scanning electron microscopy (SEM) was performed to observe the biofilm microstructure. Three samples were rinsed with PBS after 24 h, fixed in 2.5% glutaraldehyde for 2 h, and dehydrated in a graded series of ethanol (30%–100%). Isoamyl acetate was used to reach the critical point of drying, and ion sputter (108 Auto; Cressington, Watford, UK) was used for observation.

### Statistical analysis

All experiments were repeated at least three times. All analyses were performed using SPSS 18.0 statistical software (Superior Performing Software Systems, Chicago, USA). Student’s *t*-test or one-way analysis of variance (ANOVA) was performed to conduct the statistical analysis. P<0.05 was considered statistically significant.

## Results

### Construction of *E. faecalis* ∆clpP mutant strains and complement strains

**Figure 2:**
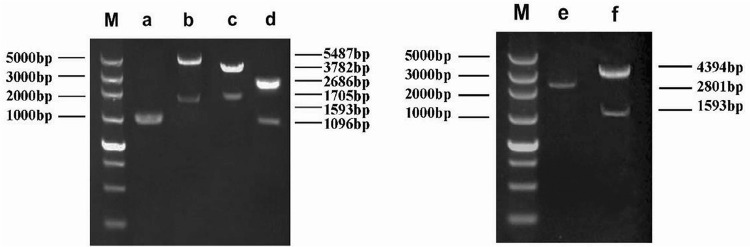
Identification of the clpP deletion strains and clpP complement strains. M: DNA marker; a) fragment of the clpP gene; b) PCR product of pUC18-∆clpP-ermB-clpP; c) PCR product of pUC18-∆clpP-ermB; d) PCR product of the pUC18-∆clpP; e) fragment of ∆clpP-ermB; f) fragment of ∆clpP-ermB-clpP

Upstream and downstream primers for the *clpP* gene were used in a PCR-based overlap extension method to obtain the *ΔclpP* fragment (1096 bp). The results are shown in Figure 2a. Next, the *ΔclpP* fragment was cloned into the plasmid pUC18 to generate *pUC18-ΔclpP*. The recombinant plasmid *pUC18-ΔclpP* was verified by PCR and enzymatic digestion, and the target bands of 1096 bp and 2686 bp were obtained (Figure 2d). The erythromycin resistance gene *ermB* was inserted into *pUC18-ΔclpP* to create *pUC18-ΔclpP-ermB*, which was verified by PstI and SmaI digestion to produce the 1705 bp and 3782 bp target bands (Figure 2c). Additionally, *clpP* was inserted into *pUC18-ΔclpP-ermB* to generate *pUC18-ΔclpP-ermB-clpP* (Figure 2b). Recombinant plasmids *pUC18-ΔclpP-ermB* and *pUC18-ΔclpP-ermB-clpP* were transformed into *E. faecalis* competent cells. After screening for positive clones, the final ∆*clpP* mutant strains and complemented strains were obtained (Figure 2e and 2f).

### ClpP protease deletion inhibited growth of *E. faecalis*

**Figure 3:**
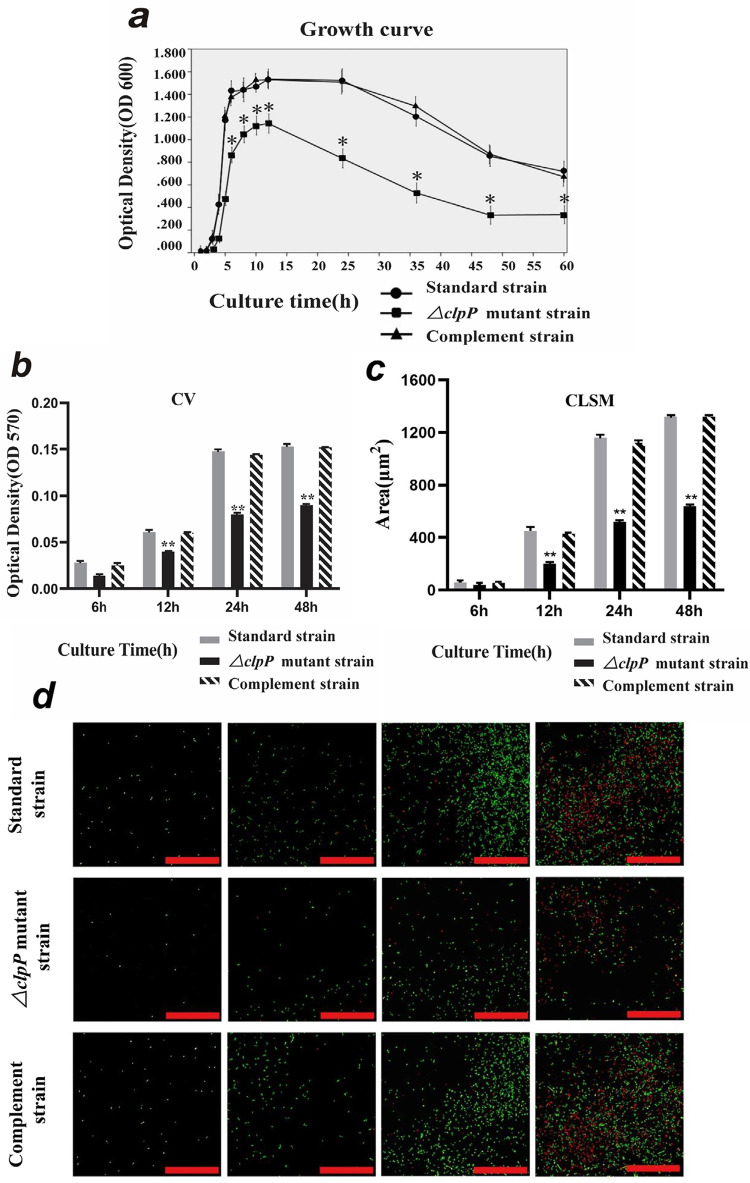
ClpP protease deletion inhibited growth and biofilm mass of E. faecalis. a) clpP gene deletion inhibits the growth of E. faecalis. The ∆clpP mutant strains exhibits impaired growth compared with the standard and clpP complement strains (p<0.05). b) Crystal violet staining shows that the biofilm mass in the ∆clpP mutant strains decreased at 12, 24, and 48 h (p<0.01) compared to the standard strains. c) CLSM examination of biofilm formation mass. The biofilm area in the ∆clpP mutant strains is smaller than that in the standard and complement strains at 12, 24, and 48 h (p<0.01). d) CLSM to observe the cell viability. The number of ∆clpP mutant bacteria increased slowly, and the proportion of dead bacteria (red) was greater than the standard and complement strains (green fluorescent signal: staining by SYT90; red fluorescent signal: staining by PI; scale bar = 50 μm)

As shown in Figure 3a, all three strains entered the logarithmic growth phase after 4 h and reached a peak at 14 h. The growth curves of the standard and *clpP complement* strains were similar. The quiescent stage was approximately 10 h–24 h, and gradually decreased thereafter. The quiescent stage of the ∆*clpP* mutant strains was 12 h–14 h, and the duration was shorter. The ∆*clpP* mutant strains exhibited impaired growth compared with the standard and *clpP complement* strains (p<0.05). This result indicates an important role for ClpP protease in the optimal growth of *E. faecalis* (Figure 3a).

### ClpP protease deletion inhibited biofilm mass

The three strains were incubated for 6, 12, 24, and 48 h for biofilm formation analysis. CV staining showed that the biofilm initially formed after culturing the strains for 6 h. With increased culture time, the biofilm mass also gradually increased. A mature biofilm was observed at 48 h. Compared with the standard strains, the biofilm mass was significantly decreased in the ∆*clpP* mutant strains at 12, 24, and 48 h. The biofilm formation ability of the complement strains was restored with no significant difference compared to the standard strains (p<0.01; Figure 3b).

### ClpP depletion inhibited cell viability

CLSM was used to determine the cell viability and biofilm mass from 6 h to 48 h. The three groups were dominated by live bacterial cells, whereas the number of bacteria in the ∆*clpP* mutant strains gradually increased with an increased proportion of dead bacteria compared to the other strains. Large biofilm sheets were observed in the standard and *clpP* complement strains cultures from 24 to 48 h; however, smaller biofilm sheets were generated by the ∆*clpP* mutant strains over the same time. These differences were statistically significant (p<0.01; Figure 3c and 3d).

### ClpP deletion reduced the polysaccharide matrix mass

**Figure 4:**
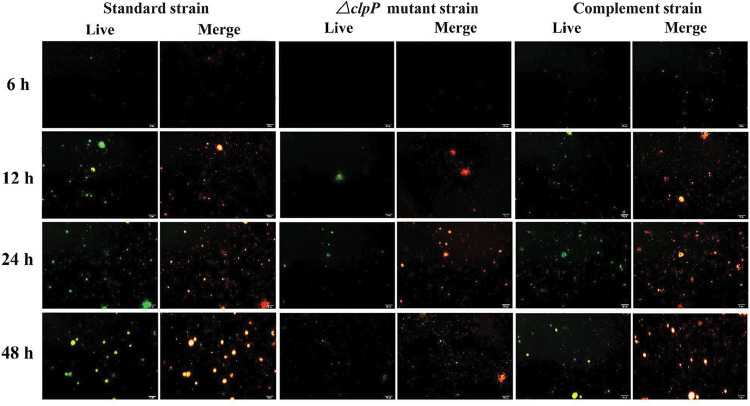
ClpP deletion reduced the polysaccharide matrix mass. Compared to the standard and complemented strains, the mass of polysaccharide matrix in the ∆clpP mutant strains were decreased at 6, 12, 24, and 48 h (Live: staining by FITC-ConA; Merge: staining by FITC-ConA and PI. scale bar = 20 μm)

CLSM detected the mass of the polysaccharide matrix to further explore the biofilm-forming capacity of the *E. faecalis* strains. Results show that the amount of polysaccharide matrix increased from 6 h to 48 h in all three strains. However, compared to the standard strains and complement strains, the mass of the polysaccharide matrix in the ∆*clpP* mutant strains was lower at all time points (Figure 4).

### Characterization of the biofilm microstructure

**Figure 5:**
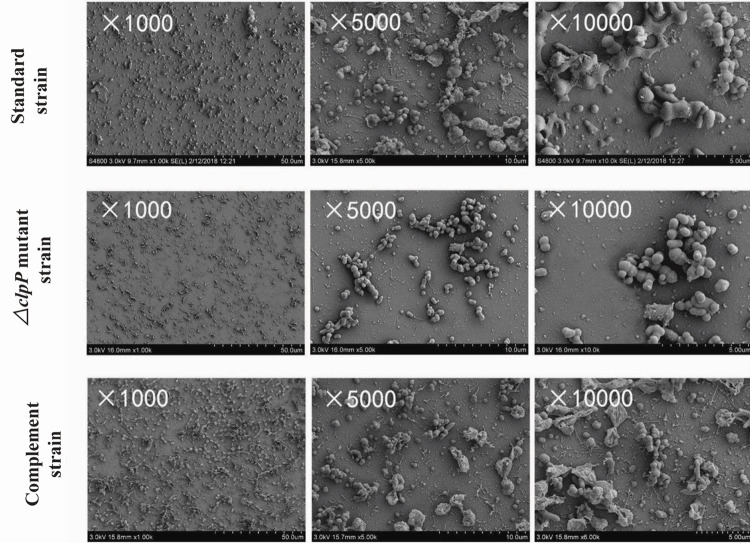
Biofilm microstructure conditions observed by scanning electron microscopy at 24 h. SEM results show that the ∆clpP mutant strains had a lower biofilm volume and a more regular shape (magnification ×1,000, ×5,000, and ×10,000)

The biofilm microstructure conditions were observed by SEM (Figure 5). After 24 h, the standard and *clpP complement* strains formed a mature biofilm structure (magnification ×1,000, ×5,000, and ×10,000) comprising grainy secretions and filaceous links with no significant difference observed between the strains. Meanwhile, the ∆*clpP* mutant strains had a lower biofilm volume and a more regular shape.

## Discussion

ClpP is a key member of the Clp family and participates in the hydrolysis of misfolded and defective proteins. For many pathogenic bacteria, such as *Listeria monocytogenes, Salmonella typhimurium, Streptococcus mutants,* and *Staphylococcus aureus*, deletion of the *clpP* gene reduces pathogenicity, indicating that ClpP plays an important role in these bacteria.^19-21^ Studies on clpP can, therefore, help us to further explore the pathogenic mechanism of various bacteria. To date, few studies have examined if ClpP participates in the survival of *E. faecalis* or enhances its ability to form biofilm.

Studies on *E. faecalis* are important for understanding the pathogenesis of biofilms, which are surface-attached microbial communities embedded in extracellular polymeric substances.^22-23^ One factor affecting the formation of biofilms is the quantity of proliferated bacteria. In our study, strains with the *clpP* gene deleted, and *clpP* complement strains were constructed using homologous recombination technology, which requires only one round of recombination to achieve gene insertion and mutation. Gene knockout enabled us to test the specific function of the *clpP* gene, which can base subsequent studies on biofilm formation. The proliferative capacity of the three strains was then observed continuously using a classic viable colony counting method, which is simple yet reliable and has a low associated cost. The results showed that the standard strains had a similar variation tendency as the *clpP complement* strains, with a quiescent stage of approximately 10 h to 24 h. However, the growth pattern of the *clpP* deletion strains was significantly altered with a shorter quiescent stage and impaired growth compared to the standard and complement strains. Moreover, the polysaccharide matrix, an important component of biofilms, was examined. Polymeric substances are not only necessary for bacterial adhesion, but also serve as a cytoskeleton in biofilm formation, which is beneficial for the survival of bacteria in this malnourished environment.^24-26^ The results show that the polysaccharide matrix mass in the *clpP* deleted strains was decreased compared with standard and complement strains.

The changes in growth pattern and the decrease in polysaccharide matrix may explain the CV and fluorescence staining results for the *clpP* deletion strains, which were examined using SEM and CLSM. These methods are effective for analyzing biofilms both quantitatively and qualitatively. The standard and complemented strains were observed to contain a large number of live bacteria during the quiescent stage (24 h). With an increased time, the biofilm area increased slowly, but the proportion of dead bacteria also significantly increased. In contrast, growth of the *clpP* deletion strains was inhibited. After the quiescent stage, the biofilm also gradually increased, but the general level was much lower than that of the standard and complement strains. It was also confirmed morphologically by SEM that mature biofilms were formed in the standard strains after 24 h, whereas the biofilm formed by the *clpP* deletion strains was loose. CLSM further showed the formation of a large biofilm sheet by the standard and *clpP* complement strains; however, only a small and dispersive biofilm was formed by the *clpP* deletion strains.

The results in our study are consistent with those observed in other Gram-positive bacteria. For example, Wang, et al.^27^ (2007) found that *clpP* influences the initial attachment of bacteria to decrease biofilm formation of *Staphylococcus Epidermidis*. This result is also consistent with our previous findings in the Gram-negative *P. gingivali*s.^15^ Studies with *Streptococcus mutans* have also reported that *clpP* gene deletion increases biofilm formation in Gram-positive bacteria under special environments.^28^ Based on these results, we hypothesized that the *clpP* gene may have different regulatory effects in different bacteria. However, a bacteria being either Gram-positive or Gram-negative does not appear to be a criterion for defining ClpP effects.

The role of ClpP in *E. faecalis* has been rarely studied. Zheng, et al.^29^ (2020) found that *clpP* inhibits the formation of *E. faecalis* biofilms, although it has no effect on the growth of *E. faecalis.* This differs from our results, which showed that *clpP* expression was also closely related to growth. Studies have also found that *clpP* influenced growth and filament formation *Salmonella Enterica Serovar Typhimurium* at low temperature.^30^Moreover, autolysis has been shown to play an important role in bacterial development, including biofilm formation.^31^ Researchers found that ClpC, another member of the Hsp100/ ATPase family, could release autolysin A to alter its growth ability.^32-33^ Further investigation is, therefore, required to determine if *clpP* can alter the proliferative capacity and biofilm formation in *E. faecalis* by autolysis.

Considering that *E. faecalis* is one of the main etiologic pathogens for root canal reinfection and persistent periapical periodontitis, whereas also exhibiting a certain resistance to most root canal therapy drugs, cleaning and disinfection methods,^34^the results of the current study may provide insights into novel targets to better control *E. faecalis* infections. Thus, we showed that ClpP can increase the bioﬁlm formation of *E. faecalis*. We also demonstrated that ClpP may serve as a potential therapeutic target for *E. faecalis*. Recently, acyldepsipeptides have been identified as a new antibiotic that targets ClpP to elicit a bactericidal role. Hence, application of acyldepsipeptides as a targeted drug for ClpP in root canal therapy could also be effective. Nevertheless, elucidation of the mechanism by which ClpP affects biofilm formation and growth of *E. faecalis* is necessary in future studies.^35^

## Conclusions

Our studied shows that ClpP can increase bioﬁlm formation in *E. faecalis* and emphasizes the importance of ClpP as a potential target against *E. faecalis*.
